# Supplementary figures and images for: Bayesian inference of admixture graphs on Native American and Arctic populations
Source: PLoS Genet. 2023 Feb 13;19(2):e1010410. doi: 10.1371/journal.pgen.1010410 (PMC9956672; doi:10.1371/journal.pgen.1010410)

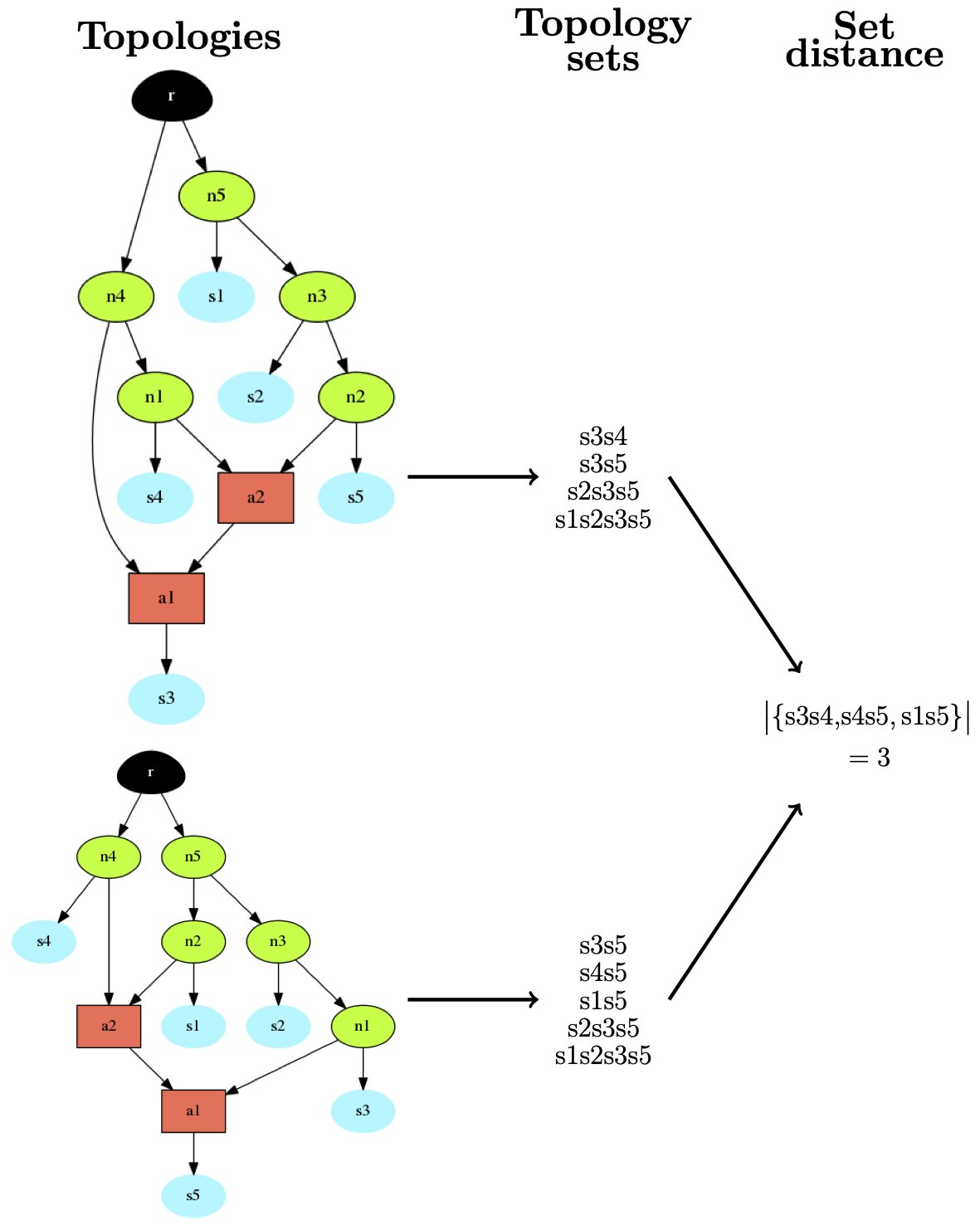

Supplement: S9 Fig — The method used to calculate the Set Distance between two admixture graph topologies (left). First, the topologies are transformed in their descendant sets/topology sets (middle). The distance is then calculated as the symmetric set distance between the two topology sets (right). (TIF) [file pgen.1010410.s009.tif]

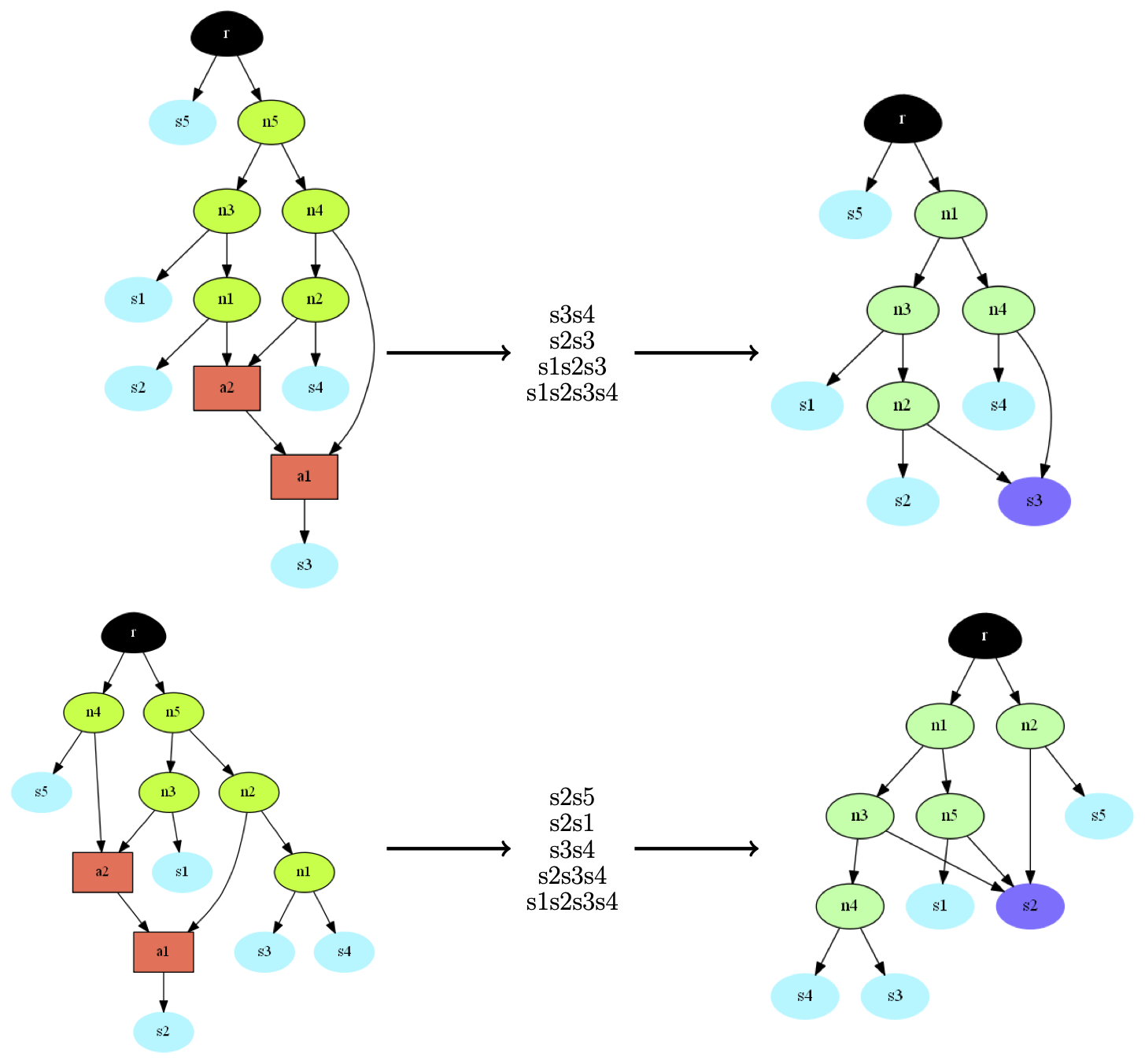

Supplement: S10 Fig — First, we derive the topology set (middle) from the topology (left). The minimal topology (right) is the smallest possible graph that is consistent with the topology set. Note, node labels assigned to the topology (left) are arbitrary and do not identify corresponding nodes in the minimal topology (right). (TIF) [file pgen.1010410.s010.tif]

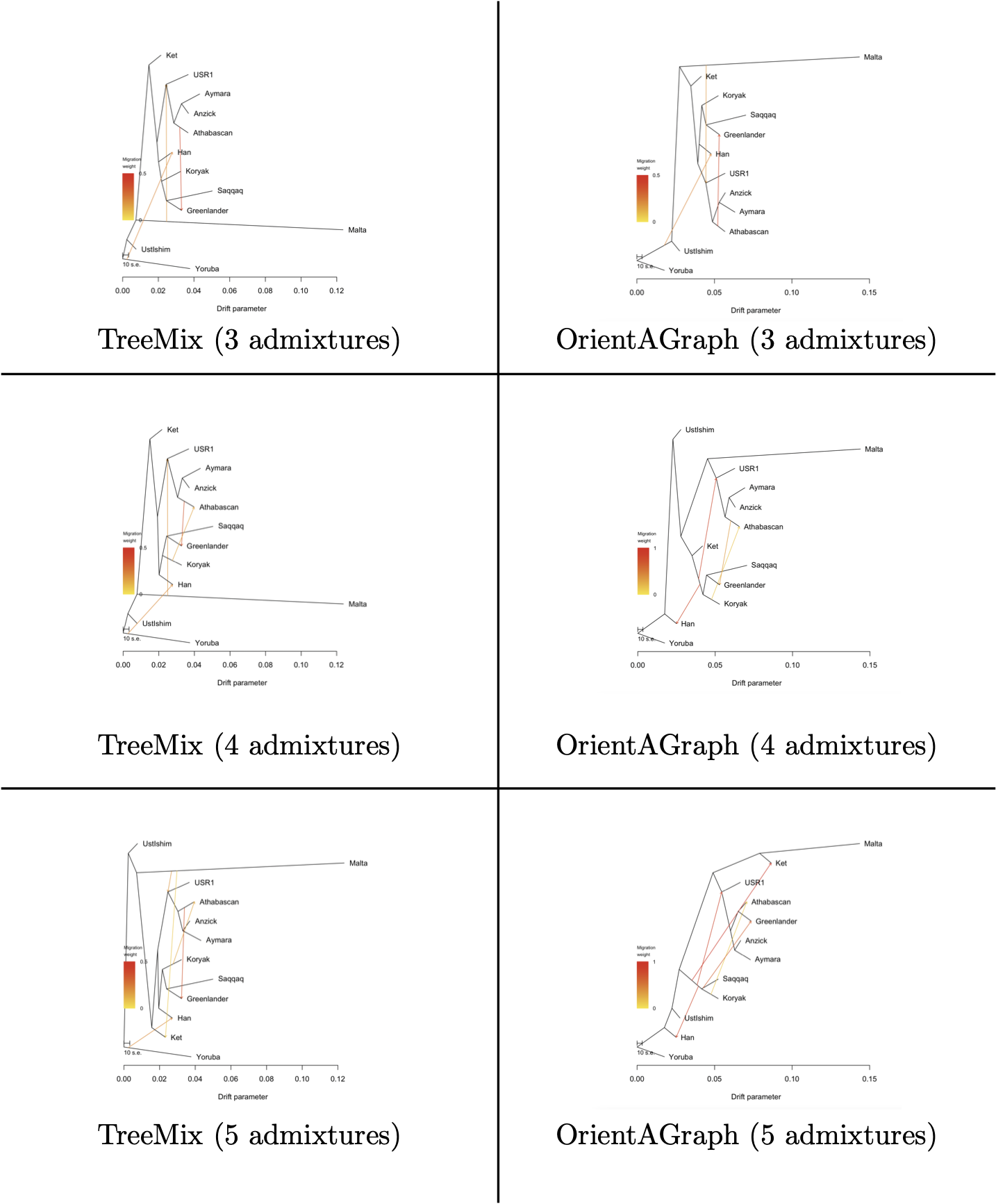

Supplement: S16 Fig — We run each method with 3, 4, and 5 admixture events as these were the numbers of admixture events in nearly all graphs sampled by AdmixtureBayes after the burn-in period (see S11 Fig). (TIF) [file pgen.1010410.s016.tif]
