# Supplementary material for: Bayesian inference of admixture graphs on Native American and Arctic populations
Source: PLoS Genet. 2023 Feb 13;19(2):e1010410. doi: 10.1371/journal.pgen.1010410 (PMC9956672; doi:10.1371/journal.pgen.1010410)
Supplement: S1 Text — (PDF) [file pgen.1010410.s017.pdf]

Supplementary Information for “Bayesian inference of admixture graphs on Native American and Arctic populations”

Contents

1 Initialization of AdmixtureBayes **2**

2 Robustness correction **3**

3 Evaluating convergence and mixing rate **5**

4 Quantifying uncertainty due to small sample sizes **6**

5 Number of admixture graph topologies **7**

1 Initialization of AdmixtureBayes

In order to use the Gelman-Rubin statistics and monitor mixing of the Markov chain, it is desirable for AdmixtureBayes to begin in a randomly chosen initial graph, ideally one that is overdispersed relative to the posterior. Unfortunately, our specification of the prior on admixture graphs does not yield a natural simulation model. We instead constructed an algorithm that simulates admixture graphs conditioned on the number of admixture events using a discrete-time Markov chain that follows lineages back in time. We sample a number of admixture events from our prior on the number of admixture events (geometric distribution with parameter 0.5, truncated to a maximum of 20) and then run the following algorithm to obtain our initial graph.

If there are L leaves, there are L free lineages at the start. Given the number of leaves and the number of admixture events, we know the number of divergence and admixture nodes. The free lineages choose a parent node uniformly at random such that

1. No more than two lineages choose the same divergence node
2. No more than one lineage choose the same admixture node
3. No 'eyes' are formed. That is, two lineages from the same admixture node will not choose the same divergence node.
4. The complete admixture graph can still be constructed. For example, if no two lineages had chosen the same divergence node, there would not be any free lineages in the next step of the Markov chain.

When two lineages have chosen a divergence node, a new free lineage is released for the next step in the Markov chain. Likewise, a chosen admixture node produces two new lineages. The algorithm stops when there is just one free lineage left and all divergence nodes and admixture nodes have been 'filled'. For topologies without admixture events, our simulation algorithm chooses uniformly among the possible topologies. For topologies with admixture events, our

algorithm inherently prefers admixture events closer to the root when compared to the uniform prior. However, each graph in the state space is still chosen with positive probability and the distribution is still overdispersed with respect to the posterior, so this is still an acceptable way of randomly choosing an initial graph for the purpose of Gelman-Rubin analysis.

2 Robustness correction

In the Bayesian phylogeny program MrBayes [1], it has been shown that independent, exponentially distributed priors on the branch lengths can unduly influence posterior estimates of total tree length [2], which could also be a problem for AdmixtureBayes. To see this, consider the average branch length $\bar{c}$. For simplicity, assume the effective population size, N_e, is constant across the admixture graph. Furthermore, suppose that the exponential rate of Eq 9 (Main Text) is 1. Let $T = \sum T_i$ be the total *time* (not drift) of all branches in the admixture graph. Then we can write

$$\bar{c} = \frac{1}{D}\sum_{i=1}^{D} e^{-\frac{T_i}{2N_e}} \approx \frac{T}{2DN_e}. \tag{1}$$

Since it is an average of independent random variables, its mean and variance are

$$E(\bar{c}) = 1 \tag{2}$$

$$\mathrm{Var}(\bar{c}) = \frac{1}{D} \tag{3}$$

This means that the prior expects $\frac{T}{2DN_e}$ to be very close to 1. However, for real datasets we would expect the ratio to vary much more, and there is no biological reason why it should be near the arbitrary number 1. For a specific dataset, if the true value of $\frac{T}{2DN_e}$ were smaller than 1, the posterior would be overestimated for admixture graphs with higher values of $\frac{T}{2DN_e}$. Such graphs would generally possess a deflated number of admixture events and thereby a

smaller D. Similarly, large true values of the ratio would result in a skew towards admixture graphs with an inflated number of admixture events.

To mitigate the problems caused by the independent, exponential priors, MrBayes includes an alternative compound Dirichlet-Gamma prior on the branch lengths, such that the variance of the average branch length can be set arbitrarily high [2]. However, we normalize the data covariance matrix and adjust the rates of the exponential distributions accordingly.

To reduce the sensitivity of our posterior estimates to the prior, we wish for the prior exponential rate of c_i to be close to $\frac{T}{2DN_e}$. We rewrite

$$E[\bar{c}] = \frac{2L-2}{D} \cdot \frac{E[\sum_i c_i]}{2L-2} \tag{4}$$

The first fraction is manageable because the prior is allowed to depend on L and D. The second fraction is the average branch length if there are no admixture events in the admixture graph. It can be estimated by summing the outgroup-leaf distances for all leaves and dividing by the number of branches between the outgroup and the leaves. Denote that divisor $\tilde{D} = \sum_{l=1}^{L} b_l$, where b_l is the number of branches between the outgroup and leaf l. We know that b_l will be 1 more than the number of branches between the root and leaf l. Unfortunately, the number of branches between the root and a given leaf node will depend on the tree topology. We therefore make the approximation $\tilde{D} \approx \sum_{l=1}^{L} (\log_2(L) + 1) = L\log_2(L) + L$ based on the fact that in a balanced full binary tree with L leaves, the number of branches between a leaf node and the root can be approximated as $\log_2(L)$. This leads to the approximation

$$E[\bar{c}] \approx \frac{2L-2}{D} \cdot \frac{E[\sum_{l=1}^{L} \sum_{i \in \mathcal{C}_l} c_i]}{L\log_2(L) + L} \tag{5}$$

where $\mathcal{C}_l$ is the set of indices of the branches between the outgroup and leaf l. Regardless of the true topology, we can estimate $E[\sum_{l=1}^{L} \sum_{i \in \mathcal{C}_l} c_i]$ by the trace

of the data covariance matrix.

$$\widehat{E}[\bar{c}] = \frac{2L-2}{D} \cdot \frac{\mathrm{tr}[S/\bar{h}]}{L\log_2(L)+L} \tag{6}$$

$$= \frac{2L-2}{D} \cdot \frac{1}{c_S} \tag{7}$$

Instead of letting (7) be the exponential rate of the branch length prior, we normalize the data covariance matrix by c_S and let $\frac{2L-2}{D}$ be the expected mean of the branch lengths. We avoid having a prior that depends on the data by moving c_S out of the prior. However, since c_S depends on the data, the matrix $c_S S/\hat{h}$ would not be Wishart distributed, even if $S/\hat{h}$ were truly Wishart distributed. The scaling by c_S therefore adds another layer of approximation to the likelihood.

This robustness correction makes the graph inference independent of the absolute scale (as measured by the trace) of the data covariance matrix. The maximum likelihood methods TreeMix [3], qpGraph [4], OrientAGraph [5], and MixMapper [6] inherently have this property as well.

3 Evaluating convergence and mixing rate

In order to evaluate the convergence of the MCMC sampler, we used two different metrics, both of which are based on examining summary statistics of the chain. The summary statistics we chose to consider were the number of admixture events, the posterior probability, and the total branch length of the graph. Our first metric was simply examining the trace plots of the chain. From these plots, it is often possible to visualize the burn-in period. The second metric was the more sophisticated Gelman-Rubin convergence diagnostic, which analyzes the behavior of several chains run in parallel from different starting states [7]. This diagnostic is based on calculating the ratio of the variance of the summary statistic between chains to the variance of the summary statistic within chains. A ratio close to 1 signifies that all chains have converged from their disparate

starting states to the same equilibrium distribution. We used the *coda* package to perform this comparison [8]. To evaluate the mixing rates of the chain, we plotted the autocorrelation of the summary statistics as a function of the lag between samples.

We demonstrated these analyses on the real dataset of Arctic and Native Americans presented in this paper. We ran AdmixtureBayes for 3 independent runs, each with `--MCMC_chains 32` (which means that each run has 32 parallel Metropolis-coupled chains which each vary in "temperature") and using a random starting state, which is the default behavior of AdmixtureBayes. We plot the convergence and mixing results in S11 Fig, S12 Fig, and S13 Fig. The exact code used to run AdmixtureBayes for this analysis as well as the convergence analysis code is available in the Convergence folder of the AdmixtureBayes GitHub.

4 Quantifying uncertainty due to small sample sizes

As discussed in the section "AdmixtureBayes Model", our method explicitly takes into account variance in the allelic covariance matrix due to sampling few haplotypes. We here give a formal demonstration of the way in which AdmixtureBayes is able to quantify uncertainty due to sampling a small number of haplotypes from each population. We consider a simple model in msprime with 4 populations whose history can be described by the simple tree (((pop1,pop2),pop3),pop4). There are no admixture events. We simulate a genomic region in this model where we sample 4 haplotypes from each population and when we sample 40 haplotypes from each population. We perform each of these simulations 100 times and run AdmixtureBayes on each of the output files (taking pop4 as the outgroup). We then examine the posterior probabilities of the inferred topologies of AdmixtureBayes. We plot the results in S15 Fig. From these boxplots, we observe that while the true topology is indeed

the one inferred by AdmixtureBayes to have the highest posterior in both cases, the simulated datasets with 40 haplotypes generate a probability distribution that is much more concentrated on the true admixture graph. We therefore see that AdmixtureBayes shows a higher level of uncertainty when sampling small numbers of haplotypes from populations and lower uncertainty when sampling many haplotypes from populations, which is consistent with the Admixture-Bayes model. The code to run this analysis is in the folder SampleSize on the AdmixtureBayes GitHub.

5 Number of admixture graph topologies

In order to compute the prior on the space of admixture graphs, we use the number of possible admixture graph topologies with K admixture events. This number grows at least exponentially with K and is further complicated by our specific requirements to the admixture graph topology. For computational convenience we will consider an extended class of admixture topologies: a *multi-graph topology* with L leaves is an acyclic directed multigraph (which is a graph that allows more than one edge between two vertices) for which

1. There exists one and only one root. That is a node with no parents and exactly one child.
2. The number of nodes with no children is L. All these nodes have only one parent and are called leaves.
3. If a node is neither a root nor a leaf, it has either
   (a) 1 parent and 2 children in which case we call it a *divergence node*, or
   (b) 2 parents and 1 child in which case we call it an *admixture node*.

This extends our original definition of an admixture graph topology by allowing eyes, i.e. admixture nodes whose parent branches merge in the same divergence node. The root is also now a node with one child instead of two, which means

that all multigraph topologies have a single branch "on top." We also ignore the outgroup. This will have no effect on the enumeration of topologies as we know it is always connected by a single edge to the root. Furthermore, we explicitly label all inner nodes. As before each admixture node will have one *main* parent branch and one *admixture* parent branch. We will use the notation

- The edges leading to leaves are referred to as *terminal edges.*
- A set of two terminal edges from a single node is a *pair.*

These graph elements are illustrated in S1 Fig.

A multigraph topology consists of a set of nodes $\mathcal{V}$, a set of main edges $\mathcal{E}_M$, and a set of admixture edges $\mathcal{E}_A$. There are L leaf nodes, $\{l_1, \ldots, l_L\} \subseteq \mathcal{V}$. For every admixture node, one of its parent branches belongs to $\mathcal{E}_A$ and the other belongs to $\mathcal{E}_M$. Note that all nodes are uniquely labeled. However, we are only interested in counting the number of topologies that differ in a nontrivial way. For example, switching the labels of leaf nodes that form a pair can be considered a trivial change to a topology. Therefore, we construct equivalence classes on the set of multigraph topologies and count those equivalence classes instead.

Let $\mathcal{E} = \mathcal{E}_A \uplus \mathcal{E}_M$ be the multiset union of $\mathcal{E}_M$ and $\mathcal{E}_A$. The admixture edges of a multigraph topology $(\mathcal{V}, \mathcal{E}_M, \mathcal{E}_A)$ are classified into two subsets, $\mathcal{E}_M$ and $\mathcal{E}_A$, but we can also disregard the classification and consider the *reduced multigraph topology* $(\mathcal{V}, \mathcal{E})$. We call a graph isomorphism between reduced multigraph topologies *shape preserving* while a graph isomorphism between multigraph topologies is *symmetry preserving.* A symmetry preserving isomorphism is clearly also shape preserving. If f is a symmetry preserving graph isomorphism, we say that f is *leaf preserving* if $f(l_j) = l_j$ for all $j = 1, \ldots, L$. When counting admixture graphs, we consider two admixture graphs different if and only if they are not isomorphic under such an isomorphism.

For a fixed number of leaves L, number of pairs P, number of admixture events K, and eyes E, we will consider the three sets

1. The set of equivalence classes under shape preserving isomorphisms is denoted $\mathcal{S}_{L,P,K,E}$. The equivalence classes are called *shapes*.

2. The set of equivalence classes under symmetry preserving isomorphisms is denoted $\mathcal{U}_{L,P,K,E}$. The equivalence classes are called *unlabeled topologies*.

3. The set of equivalence classes under leaf preserving isomorphisms is denoted $\mathcal{T}_{L,P,K,E}$. The equivalence classes are called *topologies* and sometimes explicitly *labeled topologies*.

We are particularly interested in the cardinality of the set $\mathcal{T}_{L,P,K,E}$, which we denote by $N(L, P, K, E)$. The difference between the sets $\mathcal{S}_{L,P,K,E}$, $\mathcal{U}_{L,P,K,E}$ and $\mathcal{T}_{L,P,K,E}$ is illustrated in S2 Fig.

In S2 Fig, both shapes in $\mathcal{S}_{3,1,1,0}$ correspond to two unlabeled topologies in $\mathcal{U}_{3,1,1,0}$, and each of the four unlabeled topologies in $\mathcal{U}_{3,1,1,0}$ correspond to three topologies in $\mathcal{T}_{3,1,1,0}$. However, in general some graphs exhibit more symmetry than others. Let $\mathcal{U}_S$ be the set of unlabeled topologies corresponding to the shape S, and $\mathcal{T}_U$ the set of topologies corresponding to the unlabeled topology U, so that

$$\mathcal{T}_{L,P,K,E} = \bigcup_{U \in \mathcal{U}_{L,P,K,E}} \mathcal{T}_U = \bigcup_{S \in \mathcal{S}_{L,P,K,E}} \bigcup_{U \in \mathcal{U}_S} \mathcal{T}_U \, . \tag{8}$$

As illustrated in S3 Fig, we can have $|\mathcal{U}_{S_1}| \neq |\mathcal{U}_{S_2}|$ with $S_1, S_2 \in \mathcal{S}_{L,P,K,E}$, and $|\mathcal{T}_{U_1}| \neq |\mathcal{T}_{U_2}|$ with $U_1, U_2 \in \mathcal{U}_S$, $S \in \mathcal{S}_{L,P,K,E}$.

Given an unlabeled topology $U \in \mathcal{U}_{L,P,K,E}$, choose an arbitrary multigraph topology representative of U denoted G. Let $\mathcal{T}'_U$ be the set of all multigraph topologies obtained by relabeling the L leaves of G using the $L!$ possible permutations. Clearly each equivalence class in $\mathcal{T}_U$ is represented by at least one of the elements in $\mathcal{T}'_U$, implying $|\mathcal{T}_U| \leq |\mathcal{T}'_U|$. Consider the set of elements of $\mathcal{T}'_U$ that are isomorphic to G under a leaf preserving isomorphism. It can be considered as a set of permutations, H_G, where the identity permutation corresponds to G. It is straightforward to show that H_G is a subgroup of the permutation group. Because H_G is a subgroup, its cosets are disjoint, contain the same number of

elements, and span the whole permutation group (see S4 Fig). This characterization gives us a more concrete representation of the elements $\mathcal{T}_{L,P,K,E}$, namely as equi-sized sets of permutations of the leaf-labels.

There are two basic approaches for counting phylogenetic trees with labeled leaves: recurrence by splitting the tree at the root [9] or recurrence by removal of one of the leaves [10]. The first approach is difficult to generalize to admixture graphs, but the latter strategy behaves relatively nicely. Our strategy for counting topologies is based on decomposing a topology into a recursive series of predecessors, such that we only need to count the number of possible predecessors in each step. The *predecessor* $\rho(G)$ of a labeled topology G with L leaves is defined as follows. In $\rho(G)$ the leaf l_L and the terminal edge leading to it are removed and

1) If the terminal edge was from a node with outdegree 2, the edge to it and the remaining edge from it are combined to a single edge.

2) If the terminal edge was from an admixture node, the admixture node is also removed, its parental edge in $\mathcal{E}_M$ is redirected to a new leaf l_L and its parental edge in $\mathcal{E}_A$ is redirected to a new leaf l_{L+1}.

Examples of topologies and their predecessors are given in S5 Fig. The topology with only one edge (graph $\rho(G_{1.2})$ in S5 Fig) has no predecessor. By examining the graph elements of the predecessors, we can now derive a recurrence formula

for the numbers $N(L,P,K,E)$:

$$\begin{aligned} N(L,P,K,E) = & \ 2(E+1)N(L-1,P,K,E+1) \\ & +(L-2P+1)N(L-1,P-1,K,E) \\ & +(L+2P+3K-2E-2)N(L-1,P,K,E) \\ & +\frac{2(P+1)}{L(L+1)}N(L+1,P+1,K-1,E-1) \\ & +\frac{4(P+1)(P+2)}{L(L+1)}N(L+1,P+2,K-1,E) \\ & +\frac{4(P+1)(L-2P-1)}{L(L+1)}N(L+1,P+1,K-1,E) \\ & +\frac{(L-2P)(L-2P+1)}{L(L+1)}N(L+1,P,K-1,E) \end{aligned} \quad (9)$$

The initial conditions are $N(1,0,0,0) = 1$ and $N(L,P,K,E) = 0$ if $L < 1$, $P > 2L$, $K < E$ or $E < 0$.

The predecessor of any topology in $\mathcal{T}_{L,P,K,E}$ is from one of eight possible sources $\mathcal{T}_{L',P',K',E'}$. We count $N(L,P,K,E)$ by looking at these eight sub cases and finding out which graphs in $\mathcal{T}_{L',P',K',E'}$ are eligible predecessors and of how many graphs in $\mathcal{T}_{L,P,K,E}$. An example of all the sub cases 1.1) – 2.4) is presented in S5 Fig.

1.1) The latest leaf l_L stems from an edge forming an eye in $\rho(G)$. Then $\rho(G) \in \mathcal{T}_{L-1,P,K,E+1}$, and since every topology in $\mathcal{T}_{L-1,P,K,E+1}$ has $E+1$ eyes, and every eye has two edges, the contribution to $N(L,P,K,E)$ is

$$2(E+1)N(L-1,P,K,E+1) \quad (10)$$

1.2) The latest leaf l_L stems from a terminal edge not belonging to any pairs in $\rho(G)$. Since $\rho(G) \in \mathcal{T}_{L-1,P-1,K,E}$, and every topology in $\mathcal{T}_{L-1,P-1,K,E}$ has $L-1$ terminal edges, $2(P-1)$ of which belong to a pair, the contribution to $N(L,P,K,E)$ is

$$(L-2P+1)N(L-1,P-1,K,E) \quad (11)$$

1.3) The latest leaf l_L stems from an edge belonging to a pair in $\rho(G)$. Since $\rho(G) \in \mathcal{T}_{L-1,P,K,E}$, and every topology in $\mathcal{T}_{L-1,P,K,E}$ has $2P$ edges belonging to a pair, the contribution to $N(L, P, K, E)$ is

$$2PN(L-1, P, K, E) \tag{12}$$

1.4) The latest leaf l_L stems from an edge which is neither terminal nor form an eye in $\rho(G)$. Since $\rho(G) \in \mathcal{T}_{L-1,P,K,E}$, and every topology in $\mathcal{T}_{L-1,P,K,E}$ has $2L+3K-3$ edges by induction, $L-1$ of which are terminal and other $2E$ form eyes, the contribution to $N(L, P, K, E)$ is

$$(L + 3K - 2E - 2)N(L-1, P, K, E) \tag{13}$$

2.1) The latest leaf l_L of G stems from an admixture node formed by joining together the edges l_L and l_{L+1} that form a pair in $\rho(G)$. We now have $\rho(G) \in \mathcal{T}_{L+1,P+1,K-1,E-1}$, but not every topology in $\mathcal{T}_{L+1,P+1,K-1,E-1}$ have the property $\mathfrak{p}_1$ that the leaves l_L and l_{L+1} form a pair.

Let $U \in \mathcal{U}_{L+1,P+1,K-1,E-1}$ be any unlabeled topology. By simple combinatorics, the proportion of multigraph topologies with property $\mathfrak{p}_1$ among the $(L+1)!$ elements in $\mathcal{T}'_U$ is $2(P+1)/(L^2+L)$. Since the property $\mathfrak{p}_1$ is invariant under leaf preserving graph isomorphisms, and every equivalence class under the leaf preserving graph isomorphisms in $\mathcal{T}'_U$ have the same cardinality, the proportion of $\mathfrak{p}_1$ among the labeled admixture graphs in $\mathcal{T}_U$ is also $2(P+1)/(L^2+L)$. Finally, because this applies to every $U \in \mathcal{U}_{L+1,P+1,K-1,E-1}$, using (8) we conclude that the proportion of topologies having property $\mathfrak{p}_1$ among all the topologies in $\mathcal{T}_{L+1,P+1,K-1,E-1}$ must be $2(P+1)/(L^2+L)$ too. Therefore, the contribution to $N(L, P, K, E)$ is

$$\frac{2(P+1)}{L^2+L}N(L+1, P+1, K-1, E-1) \tag{14}$$

2.2) The latest leaf l_L stems from an admixture node formed by joining together two edges belonging to two distinct pairs in $\rho(G)$. We now have $\rho(G) \in \mathcal{T}_{L+1,P+2,K-1,E}$, but not every topology in $\mathcal{T}_{L+1,P+2,K-1,E}$ have the property $\mathfrak{p}_2$ that the leaves l_L and l_{L+1} belong to two distinct pairs.

Let $U \in \mathcal{U}_{L+1,P+2,K-1,E}$ be any unlabeled topology. By simple combinatorics, the proportion of multigraph topologies having property $\mathfrak{p}_2$ among the $(L+1)!$ elements in $\mathcal{T}'_U$ is $4(P^2+3P+2)/(L^2+L)$. As before, since the property $\mathfrak{p}_2$ is invariant under leaf preserving graph isomorphisms, all equivalence classes in $\mathcal{T}'_U$ are of equal size and this holds for all unlabeled topologies, the proportion of topologies having property $\mathfrak{p}_2$ among the elements in $\mathcal{T}_{L+1,P+2,K-1,E}$ is the same. Therefore, the contribution to $N(L,P,K,E)$ is

$$\frac{4(P^2+3P+2)}{L^2+L} N(L+1,P+2,K-1,E) \tag{15}$$

2.3) The latest leaf l_L stems from an admixture node formed by joining together two terminal edges exactly one of which belongs to a pair in $\rho(G)$. We now have $\rho(G) \in \mathcal{T}_{L+1,P+1,K-1,E}$, but not every topology in $\mathcal{T}_{L+1,P+1,K-1,E}$ have the property $\mathfrak{p}_3$ that exactly one of the leaves l_L and l_{L+1} belong to a pair.

Let $U \in \mathcal{U}_{L+1,P+1,K-1,E}$ be any unlabeled topology. By simple combinatorics, the proportion of multigraph topologies having property $\mathfrak{p}_3$ among the $(L+1)!$ elements in $\mathcal{T}'_U$ is $4(PL+L-2P^2-3P-1)/(L^2+L)$. As before, since the property $\mathfrak{p}_3$ is invariant under leaf preserving graph isomorphisms, all equivalence classes in $\mathcal{T}'_U$ are of equal size and this holds for all unlabeled topologies, the proportion of topologies with property $\mathfrak{p}_3$ among the topologies in $\mathcal{T}_{L+1,P+1,K-1,E}$ is the same. Therefore, the contribution to $N(L,P,K,E)$ is

$$\frac{4(PL+L-2P^2-3P-1)}{L^2+L} N(L+1,P+1,K-1,E) \tag{16}$$

2.4) The latest leaf l_L stems from an admixture node formed by joining together two terminal edges outside the pairs of $\rho(G)$. We now have $\rho(G) \in \mathcal{T}_{L+1,P,K-1,E}$, but not every topology in $\mathcal{T}_{L+1,P,K-1,E}$ have the property $\mathfrak{p}_4$ that the leaves l_L and l_{L+1} do not belong to a pair.

Let $U \in \mathcal{U}_{L+1,P,K-1,E}$ be any unlabeled topology. By simple combinatorics, the proportion of multigraph topologies having property $\mathfrak{p}_4$ among the $(L+1)!$ elements in $\mathcal{T}'_U$ is $(L^2 - 4PL + L + 4P^2 - 2P)/(L^2 + L)$. As before, since the property $\mathfrak{p}_4$ is invariant under leaf preserving graph isomorphisms, all equivalence classes in $\mathcal{T}'_U$ are of equal size and this holds for all unlabeled topologies, the proportion of topologies having property $\mathfrak{p}_4$ among the elements in $\mathcal{T}_{L+1,P,K-1,E}$ is the same. Therefore, the contribution to $N(L,P,K,E)$ is

$$\frac{L^2 - 4PL + L + 4P^2 - 2P}{L^2 + L} N(L+1, P, K-1, E) \tag{17}$$

Formula (9) follows by summing up all the contributions (10) – (17). The recurrence procedure converges in $L + 2K$ steps, because either L decreases by one, or K decreases by one increasing L by one.

References

1. Ronquist F, Huelsenbeck JP. MrBayes 3: Bayesian phylogenetic inference under mixed models. Bioinformatics. 2003;19(12):1572-4.

2. Zhang C, Rannala B, Yang Z. Robustness of Compound Dirichlet Priors for Bayesian Inference of Branch Lengths. Systematic Biology. 2012 02;61(5):779-84.

3. Pickrell JK, Pritchard JK. Inference of Population Splits and Mixtures from Genome-Wide Allele Frequency Data. PLOS Genetics. 2012 11;8(11):1-17.

4. Patterson NJ, Moorjani P, Luo Y, Mallick S, Rohland N, Zhan Y, et al. Ancient Admixture in Human History. Genetics. 2012.

5. Molloy EK, Durvasula A, Sankararaman S. Advancing admixture graph estimation via maximum likelihood network orientation. Bioinformatics. 2021 07;37(Supplement 1):i142-50.

6. Lipson M, Loh PR, Levin A, Reich D, Patterson N, Berger B. Efficient moment-based inference of admixture parameters and sources of gene flow. Molecular biology and evolution. 2013;30(8):1788-802.

7. Gelman A, Rubin DB. Inference from Iterative Simulation Using Multiple Sequences. Statistical Science. 1992;7(4):457 472.

8. Plummer M, Best N, Cowles K, Vines K. CODA: convergence diagnosis and output analysis for MCMC. R News. 2006 March;6(1):7-11.

9. Harding E. The probabilities of rooted tree-shapes generated by random bifurcation. Advances in Applied Probability. 1971;3(1):44-77.

10. Cavalli-Sforza LL, Edwards AW. Phylogenetic analysis: models and estimation procedures. Evolution. 1967;21(3):550-70.
