# Supplementary material for: Bayesian inference of admixture graphs on Native American and Arctic populations
Source: PLoS Genet. 2023 Feb 13;19(2):e1010410. doi: 10.1371/journal.pgen.1010410 (PMC9956672; doi:10.1371/journal.pgen.1010410)
Supplement: Attachment — Submitted filename: report.pdf [file pgen.1010410.s018.pdf]

The main contribution of this paper is a reversible jump MCMC algorithm for inferring admixture graphs from DNA sequence data. Admixture graphs are canonical models of population genetics, and as the paper summarizes, existing methods tend to employ greedy search algorithms which produce a point estimate of the latent graph. The `AdmixtureBayes` MCMC method has more rigorous theoretical justification than a greedy search algorithm, and produces an ensemble of admixture graphs, facilitating uncertainty quantification. Ensembles of graphs are very valuable for robust analysis of large data sets because best-fit graphs are very unlikely to coincide with the ground truth, even in idealized cases in which it exists. The method is illustrated through several simulated and real data analyses, with comparisons to established algorithms.

The simulation study outlined on pages 6–7 demonstrates the mixing of `AdmixtureBayes` on a simulated human-like sample, but does not assess the accuracy with which the method recovers the latent admixture graph. That is not a straightforward task because the data-generating model is not an admixture graph, but I think some quantification can, and should, still be attempted. `Msprime` can be set to store migration events in the simulated ancestry using the `record_full_arg` and `record_migrations` options. Tracking the frequency and timing of migrations should yield a picture which is comparable to fitted admixture graphs output by `AdmixtureBayes`. Understanding the accuracy of the modeling framework in a simulated scenario with a reasonable degree of model error, rather than just when the data is sampled from a fixed admixture graph, would make the interpretation of real-data analyses more robust.

Some more minor points:

1. p4: "However, the Gaussian approach offers a way to compute a true likelihood..." Given that the Gaussian model is also a Brownian approximation of genetic drift, could the authors clarify what they mean by a "true" likelihood?

2. p10: "It is expected that the MAP estimate is more accurate than the average posterior graph, yet a large difference could be a sign that he sampled posterior distribution is inaccurate." I don't understand this sentence. The MAP estimate is one of the canonical definitions of "an average posterior graph". I'm also not sure what an inaccurate posterior distribution means—would this be a diagnostic for model misspecification?

3. p23: Could you briefly justify where the approximation $\tilde{D} \approx L \log_2(L) + L$ comes from?

4. p26, proposals 4–6: I would expect that the number of rejections due to negative proposals grows rapidly with the size of the underlying graph. Did you consider reflecting negative values about the origin to recover a symmetric proposal mechanism with no out-of-bounds rejections?

5. p35: Why was simulation of genetic data on admixture graphs done using `ms`, rather than `msprime`? Its `demography.add_population_split()` and `demography.add_admixture()` methods would undoubtedly be able to reproduce the simulated admixture graph, and there would be no need to cut up the genome into independent segments for reasons of computational feasibility.

6. p58, Figure S10: I think it would be useful to fix the y-axis limits within each row to make the three chains easier to compare. The log-posterior would also be easier to visualize than the posterior.

7. p59, Figure S11: Whilst it is clear that all scale reduction factors are small, I think the plot would be more informative with the y-axis truncated at, say, 1.5 rather than 3.0, to make the differences between summary statistics clearer.
