# Supplementary material for: Bayesian inference of admixture graphs on Native American and Arctic populations
Source: PLoS Genet. 2023 Feb 13;19(2):e1010410. doi: 10.1371/journal.pgen.1010410 (PMC9956672; doi:10.1371/journal.pgen.1010410)
Supplement: Attachment — Submitted filename: Admixturebayes Reviews.pdf [file pgen.1010410.s019.pdf]

Dear *PLOS Genetics* Editorial Staff,

We thank the reviewers of this manuscript very much for their helpful and constructive feedback of "Bayesian inference of admixture graphs on Native American and Arctic populations". In response to this feedback, we have made several substantial improvements to the AdmixtureBayes code and significant edits to the manuscript. The biggest changes we made were 1) A rewriting of the simulation study section that compares AdmixtureBayes to other methods. We have added a comparison to OrientAGraph and presented the results in a way we think is clearer, including changing the type of plots used to represent the results and explicitly plotting the admixture graphs we use to simulate the data. 2) A reorganization of the manuscript sections to make the manuscript easier to read and to better reflect the organization of a *PLOS Genetics* Methods paper. 3) The addition of a section quantifying the effect of small sample sizes on AdmixtureBayes behavior. This was a point that was raised by multiple reviewers and one that we felt was important enough to warrant its own section of the manuscript. 4) Uploading the VCF file for our real dataset, the R script for obtaining our allele counts file from this VCF file, and the allele counts file we use as input to AdmixtureBayes to the GitHub repository. This should make the results we present for the real data completely reproducible. 5) Uploading all simulation and analysis scripts used in this study to the GitHub repository. This should make all simulation studies and method comparisons also completely reproducible.

We also made a number of smaller edits and improvements based on reviewer comments. Below, you will find our detailed responses to each individual reviewer comment. The reviewer comments are in **black**, and our responses are in **blue**. We hope our responses are to your satisfaction and that you will consider "Bayesian inference of admixture graphs on Native American and Arctic populations" suitable for publication in *PLOS Genetics*.

Sincerely,

Andrew Vaughn

Corresponding author, "Bayesian inference of admixture graphs on Native American and Arctic populations"

Reviewer 1

The main contribution of this paper is a reversible jump MCMC algorithm for inferring admixture graphs from DNA sequence data. Admixture graphs are canonical models of population genetics, and as the paper summarizes, existing methods tend to employ greedy search algorithms which produce a point estimate of the latent graph. The AdmixtureBayes MCMC method has more rigorous theoretical justification than a greedy search algorithm, and produces an ensemble of admixture graphs, facilitating uncertainty quantification. Ensembles of graphs are very valuable for robust analysis of large data sets because best-fit graphs are very unlikely to coincide with the ground truth, even in idealized cases in which it exists. The method is illustrated through several simulated and real data analyses, with comparisons to established algorithms.

The simulation study outlined on pages 6–7 demonstrates the mixing of AdmixtureBayes on a simulated human-like sample, but does not assess the accuracy with which the method recovers the latent admixture graph. That is not a straightforward task because the data- generating model is not an admixture graph, but I think some quantification can, and should, still be attempted. Msprime can be set to store migration events in the simulated ancestry using the record full arg and record migrations options. Tracking the frequency and timing of migrations should yield a picture which is comparable to fitted admixture graphs output by AdmixtureBayes. Understanding the accuracy of the modeling framework in a simulated scenario with a reasonable degree of model error, rather than just when the data is sampled from a fixed admixture graph, would make the interpretation of real-data analyses more robust.

Thank you for this comment. We have done a major rewrite of the simulation study results. In our new simulation study section, we evaluate the accuracy of AdmixtureBayes with respect to its ability to recover a set of latent admixture graphs. In particular, we simulate data from a set of admixture graphs in msprime, run AdmixtureBayes on this data, in addition to the methods TreeMix and OrientAGraph, and measure the accuracy of the inferred admixture graphs with respect to the true admixture graph that was used to generate the data. The new section is labeled "Comparisons with TreeMix and OrientAGraph".

Some more minor points:

1. p4: "However, the Gaussian approach offers a way to compute a true likelihood. . . " Given that the Gaussian model is also a Brownian approximation of genetic drift, could the authors clarify what they mean by a "true" likelihood?

   You are correct. The Gaussian model is indeed an approximation. We meant to stress the desire to compute a likelihood, rather than a pseudolikelihood, even if we must make an approximation to that likelihood. We have clarified this in the main text to make it clear we are still making an approximation. The sentence now reads: "However, the Gaussian

approach offers a way to instead approximate a true likelihood (rather than a pseudolikelihood), which we use in this paper."

2. p10: "It is expected that the MAP estimate is more accurate than the average posterior graph, yet a large difference could be a sign that he sampled posterior distribution is inaccurate." I don't understand this sentence. The MAP estimate is one of the canonical definitions of "an average posterior graph". I'm also not sure what an inaccurate posterior distribution means—would this be a diagnostic for model misspecification?

   We have made significant edits to the way we present the simulation study section and this sentence no longer exists. The new paragraph that begins "For each of the 4 admixture graphs we analyzed…" contains an explanation of how we do our mode and mean posterior graph analysis.

3. p23: Could you briefly justify where the approximation $\tilde{D} \approx L \log2(L) + L$ comes from?

   We have added an explanation of this to the "Robustness Correction" section. This approximation comes from the fact that the distance from a leaf node to the root node in a balanced full binary tree with L leaves can be well-approximated by $\log_2(L)$. We have added a sentence that reads: "We therefore make the approximation D = $\widetilde{D} \approx \Sigma_{l=1}^{L}(log_2(L) + 1) = L\ log_2(L) + L$ based on the fact that in a balanced full binary tree with L leaves, the number of branches between a leaf node and the root can be approximated as $\log_2(L)$."

4. p26, proposals 4–6: I would expect that the number of rejections due to negative proposals grows rapidly with the size of the underlying graph. Did you consider reflecting negative values about the origin to recover a symmetric proposal mechanism with no out-of-bounds rejections?

   An excellent suggestion. We have implemented boundary reflection for proposal 4, the random walks on the branch lengths. While we have not noticed any problems with mixing in the examples presented in this paper, we agree that this would almost certainly create a high number of rejections in graphs with larger numbers of edges. We have also implemented boundary reflection for proposal 6, even though this proposal does not scale with graph size. For proposal 5, we could in theory implement a boundary reflection mechanism to ensure no out-of-bounds rejections. It is slightly more complicated as we would have to reflect arbitrarily many times, as there are 2 boundaries. We attempted this but instead found that the most efficient thing to do is simply to sample all admixture proportions from the standard uniform distribution. In other words, the proposed admixture proportions will be independent of the current admixture proportions and there

is now no adaptive parameter for this step. We have updated both the manuscript and code on the GitHub accordingly.

5. p35: Why was simulation of genetic data on admixture graphs done using ms, rather than msprime? Its demography.add population split() and demography.add admixture() methods would undoubtedly be able to reproduce the simulated admixture graph, and there would be no need to cut up the genome into independent segments for reasons of computational feasibility.

   Thank you very much for this comment. We have done a major reanalysis and rewrite for the simulation study section. We now use the population_split and add_admixture methods in msprime and do not cut up the genome into regions. We have uploaded all relevant simulation scripts to the AdmixtureBayes GitHub.

6. p58, Figure S10: I think it would be useful to fix the y-axis limits within each row to make the three chains easier to compare. The log-posterior would also be easier to visualize than the posterior.

   We have fixed the y-axis limits within each row. What we call the posterior in the plot is actually the log-posterior. We have changed the y-axis label to clarify this.

7. p59, Figure S11: Whilst it is clear that all scale reduction factors are small, I think the plot would be more informative with the y-axis truncated at, say, 1.5 rather than 3.0, to make the differences between summary statistics clearer.

   We have changed the scale of the y-axis, so it is truncated at 1.5.

Reviewer 2

S. Nielsen et al. have developed a novel method for characterizing admixture graphs. The authors adapt an existing allele frequency covariance approach and add MCMC statistical machinery that allows a more rich characterization of admixture graphs compared to existing methods that mostly focus on estimating a single best, admixture graph.

I agree that searching for the best topology, or a set of reasonable ones, for an admixture graph is a real problem, as existing methods are not particularly well suited to finding the set of high-probability admixture graphs. These methods often require extensive direct input and subjective judgment, making them difficult to apply in a consistent manner. The authors also present a small suite of topological-based methods for summarizing admixture graphs that are potentially useful moving forward.

The authors apply the new method to simulated data, compare the results with an existing related method, Treemix, and apply the method to a set of 11 population samples with a goal of better

understanding the genetic relationship between Koryak, Saqqaq, Athabascans, and native Greenlanders.

It is a substantial amount of work to develop a MCMC procedure to efficiently sample the huge space of possible admixture graphs. The authors present a set of 7 proposals for alterations to graphs that seem to be able to move between possible graphs in a way that respects detailed balance. It is difficult to evaluate the extent that the full space of possible graphs is explored, but I will assume it is. Towards this goal, the authors use an adaptive Metropolis-Coupled MCMC that attempts to better explore multimodal distributions.

While I found the text generally clear, I found the structure and flow of the manuscript challenging. PLOS Genetics prescribes a Results/Discussion/Methods format. I understand this structure has the potential to make it difficult to find the best way to present Methods-focused papers, but I think the manuscript would benefit from reorganization.

For example, the results section starts with an evaluation of MCMC chains, while this is an important aspect of conducting any MCMC analysis, it is not really the main focus of the current study, and no results are actually presented in the text. It is followed by a comparison to Treemix section that spends 1.5 pages describing the methods for the comparison. Again this is important, but should not precede the basic description of AdmixtureBayes. Main figures 3, 4, 5, 6, 7 are only referenced in the appendix, and all figures applying the method to data (simulated or real) are supplemental figures. This is out of step with the title that highlights applying this method to real data. "... on Native American and Arctic populations".

You make an excellent point about the placement of main vs. supplemental figures. We have rearranged the placement of many figures.

I suggest the Results section should cover the following topics in order - 1) Method introduction, justification and important details, 2) Evaluation on simulated data / comparison to other methods 3) Application to real data. This would better match other papers in the field, such as the Treemix paper, also published in PLOS Genetics. This also better matches the alternative formatting for Methods papers at: https://journals.plos.org/plosgenetics/s/submission-guidelines.

The Methods section can supplement this formatting, by starting with e.g., 1) a full description of the method, 2) Description of simulations / comparisons to other methods, 3) real data sets. Self-contained details of specific aspects can be moved to an appendix or supplement.

Thank you for these comments on manuscript organization. We have made several edits to the organizational structure of this paper. We have moved the convergence and mixing analysis to the appendix. The Results section now begins with an introduction to our formal definition of an admixture graph as well as a textual description of the AdmixtureBayes algorithm. The Results section continues with a description of the results on simulated data and method comparison, followed by our results for the real data. The Methods section contains the full mathematical description of the method (including the likelihood specification, prior, and MCMC framework),

followed by the summaries we use to evaluate inferred graphs, a detailed description of our method comparison framework, and a description of the real data set. We have moved several sections such as the Robustness Correction to the Appendix to avoid cluttering the main text.

I was able to download and install AdmixtureBayes from the provided github link and to run an analysis on the provided example data file without any problems. My strong suggestion for the software would be to utilize a common data format, such as VCF, rather than a file format unique to a particular program. Also I might suggest splitting step 1 in the analysis outline into two steps, 1) estimation of the population covariance matrix and 2) run the MCMC. I did not see any built-in way to estimate or examine the covariance matrix.

Thank you for these suggestions. It is not directly possible to use a VCF as the input to AdmixtureBayes as there is not a 1-to-1 correspondence between individuals in the VCF file and populations in AdmixtureBayes. In other words, the user must always specify the mapping between individuals and populations. However, we have added a sample R script "ConvertFromVCF.R" that could be useful as a template for this task as well as a sample VCF "sampleData.vcf" to the AdmixtureBayes GitHub. We have also added an option to Step 1 of AdmixtureBayes to allow the covariance matrix to be saved in a separate file for examination. As described in the main text, the actual covariance matrix used by AdmixtureBayes is a scaled, bias-corrected transformation of the naïve covariance matrix that would be suggested by Equation 4. It is this matrix transformation that is saved to a file. We have made this clear in the method documentation on the GitHub page.

Small issues:

The authors show how small numbers of sampled haplotypes can reduce the accuracy of estimated admixture graph topology (figure S2). However, it was not immediately clear if AdmixtureBayes results accurately characterize the uncertainty due to sampling few haplotypes. This seems crucial to interpreting the results applying AdmixtureBayes to real data, such as in the current manuscript.

Thank you for this comment. We agree that understanding this is crucial to interpreting AdmixtureBayes output. We have added a new subsection called **Quantifying uncertainty due to small sample sizes** where we explicitly show how AdmixtureBayes characterizes uncertainty due to sampling few haplotypes. We take a specific model in msprime and simulate 100 datasets with 4 haplotypes sampled per population and 100 datasets with 40 haplotypes sampled per population. We observe that while the true topology is indeed the one inferred by AdmixtureBayes to have the highest posterior in both cases, the simulated datasets with 40 haplotypes generate a probability distribution that is much more concentrated on the true admixture graph. We therefore conclude that AdmixtureBayes correctly characterizes uncertainty due to sampling small numbers of haplotypes from populations.

Due to a lack of background, I was not able to adequately review the topological arguments present in Appendix A.3, (the justification of the flat prior on the topology). This could be split off into a separate manuscript to be submitted to another journal such as Theoretical Population Biology, or another appropriate journal where it could stand on its own and get a separate review.

Thank you for this suggestion. After a thorough discussion among all the authors, we have come to the conclusion that splitting this section off into a separate manuscript would not be the best idea. The reason for this is that we consider the enumeration of admixture topologies to be essential to this manuscript. As AdmixtureBayes performs Bayesian inference, it is critical that the prior be properly designed and clearly stated. As the prior on admixture topologies depends on the number of topologies with a given number of leaves and number of admixture events, we feel that it is important for the section enumerating the number of such topologies to be included in this manuscript, as it currently is.

The application of AdmixtureBayes to the real data set and the accompanying discussion did a good job highlighting how often researchers have specific questions, such as "is there admixture present?", "what is the source of admixture in this population?", etc. As a suggestion it could be useful to examine different types of relationships between populations, and how well they are characterized by AdmixtureBayes. Currently this is all done in figure S3, using the mode set distance statistic, which is not so easy to see how to interpret biologically.

Thank you for this comment. One application of AdmixtureBayes is indeed the ability to answer specific questions. Our primary way of addressing this is by providing functionality for analyzing graphs induced by a subset of nodes. The mode set distance is not a metric for comparing relationships between populations. Rather, it is a metric to measure the accuracy of inferred graphs relative to the true graph. AdmixtureBayes allows you to print out the top sampled admixture graph topologies along with their posterior probabilities through the "--write_rankings" option to the plotting functions. With this in hand, the user is able to obtain the posterior probabilities of any particular question they may have, including the presence or absence of a particular admixture event.

It was difficult to find the number of simulation and analysis replicates that the simulation and Treemix comparison results were based on.

We have done a major rewrite of the simulation study results and have presented it in a way we think is clearer. In addition, we have uploaded all relevant simulation scripts and AdmixtureBayes, TreeMix, and OrientAGraph commands to the AdmixtureBayes GitHub. We have listed the name of the relevant GitHub folders in the manuscript where appropriate. We hope that this makes the simulation study portion of the paper easier to understand and entirely reproducible.

The code for the two simulation evaluations are nice to include in the manuscript, but a higher level explanation of the demography, as well as a statement of why this is an appropriate demography / admixture graph would aid understanding of the results.

Thank you for this comment. We have done a major reanalysis and rewrite for the simulation study section. We have changed the convergence analysis section so the data we are analyzing is the real Arctic and Native American dataset we analyze in the Results section. This is clearly an appropriate graph to analyze as it is the real data we analyze in the results section. Furthermore, it helps show that the results we present reflect a converged, well-mixed Markov chain. For the section on comparisons with other methods, we now choose a few admixture graphs to analyze, including several previously studied admixture graphs. We have provided relevant citations for these graphs when appropriate and have included figures of the graphs.

Page 4- "It has previously been used in several other methods aimed at modeling the joint distribution of allele frequencies among populations" please support with citations.

We have supported this in the manuscript with additional citations.

Page 7 - "We then ran AdmixtureBayes for three different chains, each with --MCMC chains 16" is this 3, 16 or 48 chains?

We have clarified this in the manuscript (and changed the dataset we are running this analysis on). AdmixtureBayes is called 3 separate times. During each call, there are 32 parallel chains run in the standard Metropolis-coupled MCMC framework. Each of these chains has a different temperature, where the coldest chain is the chain from which we wish to sample. This sentence now reads: "We ran AdmixtureBayes for 3 independent runs, each with --MCMC_chains 32 (which means that each run has 32 parallel Metropolis-coupled chains which each vary in ``temperature") and using a random starting state, which is the default behavior of AdmixtureBayes."

Page 19 - Admixture graphs are described here, and this could be useful if the rest of the paper utilizes this description. However, it is not clear how to match the description here with the example admixture graph present in Figure 1. This description states "There exists one and only one root. That is a node with no parents and exactly two children". Yet, to my eye, Figure 1 contains zero nodes with no parents and two children.

We recognize that this was confusingly explained. In our definition of an admixture graph, we have now explicitly made clear what the outgroup is and how it is connected to the root node by an edge. We have also made it clear in the "Number of admixture graph topologies" section that we ignore the outgroup when computing the number of admixture topologies. This is acceptable as the presence or absence of the outgroup has no effect on the enumeration of admixture graph topologies.

Page 35 - It would help to provide a figure of the admixture graph that is simulated by this code.

Thank you for this comment. We have done a major reanalysis and rewrite for the simulation study section. For the section on comparisons with other methods, we now choose a few admixture graphs to analyze, including many previously studied admixture graphs. We have included figures of the graphs.

Figures

In general I found the figures under-described, with many explanations not repeated on each relevant figure. Many main figures were not referenced in the main text, but I appreciated the figure representing topological concepts. In general, I thought the supplemental figure were better suited for main, while most main figure were better suited to the supplement.

S1 - Should be moved to main. Reorder the subplots so similar statistics are next to each other - eg . (mode, mean) Topology equality, (mode mean) set distance.

We have done a major rewrite of the simulation study section. This particular figure no longer exists. However, our new way of representing the results of our simulation study does indeed plot similar statistics next to each other. This new figure is included in the main text.

S3 - why was only this statistic out of the 4 based on topology selected to be shown.

We have done a major rewrite of the simulation study section. This particular figure no longer exists. For the new simulations, all topology and covariance statistics are used.

S4 - why are the two subfigures different sizes? Node labeling could be improved as it is difficult to distinguish the support from the node label. Please explain coloring in the figure legend.

We have resized these subfigures and explained the node coloring in the figure caption. These figures are automatically generated by AdmixtureBayes and use the internal node labeling that is inherent to the algorithm. For consistency with the output of the algorithm and between all plots presented in this paper, we have kept the node labeling the same. However, we have added a caption for this figure that explains the node labeling, which should help distinguish the support from the node label.

S6 and S7 I think these plots are good examples of the additional utility possible with AdmixtureBayes. However in this presentation I did not understand how the posterior of the interior nodes combined with the posterior for the topology e.g. the ((Koryak, Saqqaq), Athabascan) tree.

We have added an explanation to the caption of this figure which we think explains the difference between the posterior of the topology and the posterior of each node.

Reviewer 3

PGENETICS-D-22-01018

"Bayesian inference of admixture graphs on Native American and Arctic populations"

In this study, the authors evaluated the ability of admixture graphs to reconstruct population relationship in the Americas based on genome data. They present a new reversible jump MCMC algorithm for sampling high-probability admixture graphs and show that this approach works well both as a heuristic search for a single best-fitting graph and for summarizing shared features extracted from posterior samples of graphs. They subsequently used this method with data from 11 Native American and Siberian populations to address the relationship between Saqqaq, Inuit, Koryaks, and Athabascans. In contrast with previous work, they find that "the Saqqaq is not a good proxy for the previously identified gene flow from Arctic people into the Na-Dene speaking Athabascans."

From my assessment of the admixture graph analysis, the authors' analysis seems robust, and has been directed at issues concerning optimality and likelihood issues arising from the use of other admixture graphing methods commonly employed in population genetics studies.

Yet, having followed the development of admixture graphs over a number of years, I would like to pose some questions to the authors about their use for modeling the peopling of the Americas based on ancient and modern genome data.

From an admixture standpoint, how accurately can a single genome represent a "population"? Can the Saqqaq individual really represent an entire ancestral group? What is the consequence of adding more individuals per population when estimating admixture graphs? Can this effect be modeled in order to determine how population level data (e.g., 20-30 individuals) will affect the accuracy of these estimates? How much certainty can we place on the results of admixture analysis with ancient and modern individuals when data from key ancient populations are lacking, i.e., those ancestral to modern groups which now occupy certain regions of northeast Asia or North America?

These are germane questions for efforts to reconstruct the peopling of the Americas, when initial population movements into North America may have been pulsatile in nature and those occurring in Beringia during the latter stages of the LGM may have been quite dynamic.

You raise an excellent point about the ability of a single genome to represent a population and about whether we can measure the improvement that could be achieved by using population level data. The mathematical formulation of AdmixtureBayes explicitly incorporates uncertainty due to small numbers of haplotypes. We demonstrate this explicitly by adding a new section to the paper called **Quantifying Uncertainty Due to Small Sample Sizes** where we explicitly show how AdmixtureBayes characterizes uncertainty due to sampling few haplotypes. We take a specific model in msprime and simulate 100 datasets with 4 haplotypes sampled per population

and 100 datasets with 40 haplotypes sampled per population. We observe that while the true topology is indeed the one inferred by AdmixtureBayes to be most likely in both cases, the simulated datasets with 40 haplotypes generate a probability distribution that is much more concentrated on the true admixture graph. We therefore conclude that AdmixtureBayes correctly characterizes uncertainty due to sampling small numbers of haplotypes from populations.

We also recognize that there may be some key ancient populations for which we do not have data. Our results could change if samples from these populations are used, as well as if further modern populations are considered. We make an explicit note of this in the **Discussion** section.

On this same note, modeling the relationships between circumarcric populations is not necessarily easy. Greenlanders are not precisely the same group as Alaskan Inuit, Yupik, Aluttiq or Aleut populations based on language and culture, which took hundreds if not thousands of years to evolve. The same thing is true to some extent with genetic data, which suggests subtle differences between Eskimoan-speaking groups and more substantial ones between these groups and Aleuts and Athapaskans (Na-Dene). The Athapaskans used in this study also represent one only group of Na-Dene speakers whose homeland may be Southeast Alaska and from which they spread into what is now Alaska and Canada.

In this regard, the authors seem to indicate that they consider Athapaskans to be "Native Americans" inclusive of Amerindians (the first wave of settlers). Is this the case or is the term "Native American" being used here simply to distinguish between Eskaleut-speaking groups and all other indigenous population of the Americas?

It is true that with the wide geographic and temporal span of our data, we sometimes had difficulty creating group labels that referred to a set of distinct populations. In this context, we use the term Native American to refer to non-Eskaleut-speaking indigenous populations of the Americas. We acknowledge that this is not necessarily a perfect definition, as it includes many culturally and linguistically distinct groups. In this manuscript, we use the specific population name when possible and use Native American to refer to non-Eskaleut-speaking indigenous populations of North and South America when such a labeling is necessary.

I do find it intriguing that the authors' analysis suggests that that Athapaskans are best represented as "the result of admixture between a Native American population and a Siberian population most closely related to the Koryak, but not the Saqqaq." However, previous studies have produced Y-chromosome data linking circumarctic groups including the Saqqaq and Koryaks, and the Saqqaq also show affinities with the Nganasan, Koryak, and Chukchi of northeastern Siberia based on autosomal data. In light of these findings, how can we explain the admixture results presented by the authors?

You are indeed correct that previous studies have shown that there is a strong affinity between the Koryak and the Saqqaq. We do not dispute this, and we believe the results we present do not contradict this. Indeed, the Koryak and Saqqaq are located very close together in our inferred

admixture graphs. Our claim is merely that the Siberian introgression into the Athabascan lineage is *best* represented by the Koryak, rather than the Saqqaq. This can be true without contradicting the fact that the Saqqaq and Koryak have a high degree of shared ancestry.

While recognizing that this manuscript has been submitted to a genetics paper, it is important to reconcile genetic admixture studies such as this with other archaeological, ethnographic, genetic and linguistic evidence to 'ground truth' the results of the modeling work. For this reason, I would be most interested to know the authors' perspective on these issues.

You are of course correct that the field of genetics must work in collaboration with other diverse fields in order to obtain an accurate picture of the historical movement of different people groups. However, there is still a high degree of disagreement over several fundamental questions in this field, even when considering genetic data alone. We view this paper as a contribution towards clarifying the genetic evidence by improving on algorithms for admixture graph inference that give incorrect results. We recognize that this correction of methods for genetic analysis may not necessarily provide insight into the linguistic or archaeological data, but we still consider it to be an important contribution to the field. We add several sentences to the Discussion that address this.

Reviewer 4

Here, Nielsen et al (2021) present a Bayesian method for estimating admixture graphs from allele frequency data. Their approach AdmixtureBayes is evaluated on simulated and real data sets. The results on simulated data sets are promising, and the results on biological data (on "peopling of the Americas") is quite interesting as well (although I am not a domain expert). Overall, this paper makes a strong contribution to the literature on admixture graphs, and I expect the AdmixtureGraph methodology to be of interest to method developers and method users alike.

Nevertheless, this paper could be improved in several ways. I make the following recommendations.

(1) The biological data set should be made publicly available (specifically the authors should provide the VCF and allele frequencies rather than just the accession IDs for genomes).

An excellent point. We have created a new folder on the AdmixtureBayes GitHub repo called "Data". This folder contains the VCF file for the data, the processed allele count file to be used as input to AdmixtureBayes, as well as an R script for obtaining the allele count file from the VCF file.

(2) The comparison study should include an additional method (their current comparison is against TreeMix but a new method OrientAGraph has been shown to improve the topological accuracy of TreeMix).

Thank you for alerting us to the OrientAGraph method. We have added a comparison to OrientAGraph to the manuscript.

(3) There are some figures that should be updated so that readers can more quickly understand the differences between running TreeMix and AdmixtureGraph on simulated and biological data sets.

We have made a major update to the way that we plot the results of our simulation analysis. We think the results are now easier to interpret.

Details are below.

#1. Data Availability

This is a major area of research with new methods being developed each year. A limiting factor to comparing methods and developing new methods is that many studies do not publish their data in a usable form. Often only acession IDs for genomes are provided, even though the developed methods require VCFs or allele frequencies as input.

+1a. It looks like Nielsen et al (2021) currently only provide a link to the original Nature paper (which in turn only provides accession IDs). I strongly recommend that Nielsen et al (2021) to make their processed data (i.e., VCF and allele frequencies) publically available. This will make their results reprocible and will help future studies, increasing the impact of this paper. (of all of my comments, this is the one that I feel is the most important to address)

An excellent point. We have created a new folder on the AdmixtureBayes GitHub repo called "Data". This folder contains the VCF file for the data, the processed allele count file to be used as input to AdmixtureBayes, as well as an R script for obtaining the allele count file from the VCF file.

+1b. It's great that Nielsen et al (2021) make the msprime commands available in the paper so that future researchers can benchmark methods on the simulated data sets related to this study (they go above and beyond and provide the ms command as well). I encourage the authors to upload the simulated data sets themselves to Github or some other platform.

This is a good suggestion, and we have followed it when possible. However, the total size of all the simulated datasets for our method comparison section is quite large (tens of gigabytes). This

is prohibitively large for uploading to GitHub. However, we have now added a folder on the AdmixtureBayes GitHub (ManuscriptSimulations) that contains all relevant simulation and analysis scripts used in the manuscript, which should make the results (including dataset generation) entirely reproducible.

#2. Discussion of and comparison to existing methods

+2a. Nielsen et al (2021) discuss existing admixture graphs methods on pages 3-4, focusing on well-established methods, like TreeMix (2012), qpGraph (2012), and MixMapper (2013). I recommend discussing some more recently developed methods, like miqoGraph (2021) and OrientAGraph (2021), in addition.

Please see our responses to comments 2d and 2e.

+2b. Nielsen et al (2021) write on page 4 "TreeMix searches through many potential admixture graphs without user input." I recommend changing this sentence to read something like "TreeMix searches through potential admixture graphs without user input by way of an efficient greedy heuristic." This would tie into the comments on page 4 about the downsides of greedy algorithms (where they fail to effectively search network space, getting trapped in local optima).

Thank you for the comment. We have changed this sentence to the sentence you suggested.

+2c. I recommend that Nielsen et al (2021) include the command used to run TreeMix on page 37ish. It could also be interesting to note that their observation (that changing the seed doesn't impact the graph topology recovered by TreeMix very much) was also reported by Molloy et al (2021), although these authors varied the population addition order explicitly rather than the seed.

We have uploaded all relevant simulation scripts and AdmixtureBayes, TreeMix, and OrientAGraph commands to the AdmixtureBayes GitHub. We have also listed the name of the relevant GitHub folders in the manuscript where appropriate. In the manuscript, we also have included the observation that Molloy et al (2021) also found that changing the random seed/input order of TreeMix does not affect the inferred topology.

+2d. I recommend that Nielsen et al (2021) add OrientAGraph to their comparison study because OrientAGraph has been demonstrated to produce graphs that are as accurate as those produced by TreeMix or else more accurate. This comparison should be relatively easy to add because OrientAGraph was shown to be negligibly slower than TreeMix on a data set with 10 populations and 2 admixture graphs. Moreover, OrientAGraph takes the same input as TreeMix (allele frequencies) and has nearly the same command. The only difference is the addition of -mlno 1,2 and -allmigs 1,2 flags (where 1,2 indicate that the admixture graph topology search should be expanded with the MLNO and ALLMIG algorithms after adding the first gene flow edge and after adding the second gene flow edge).

Thank you for alerting us to the OrientAGraph method. We have added a comparison to OrientAGraph to the manuscript (we indeed find that OrientAGraph has superior or equal performance to TreeMix) and added the scripts containing the OrientAGraph commands to the GitHub.

+2e. It would be great to add a comparison to miqograph as well but this method is more difficult to run in my experience because it relies on Gurobi and the output of the admixture graph is in a non-standard format.

Thank you for this suggestion. However, we examined the miqograph method in detail, and we have decided that a comparison with miqograph is not something we think will be helpful for this manuscript. We have several reasons for this. Firstly, miqograph is only able to infer admixture events that happen at the leaf nodes, a major topological limitation. While we could simulate an admixture graph with a deep admixture event and show that miqograh is unable to recover the latent graph, it is unclear that this will generate any new insight. In addition, the branch lengths and admixture proportions of miqograph are fixed at discrete numbers, rather than allowed to vary continuously. In summary, the graph space explored by miqograph is very different from the graph space explored by AdmixtureBayes. Secondly, the Gurobi software is proprietary, and it would therefore be very difficult for the results of any miqograph comparison we present to be replicated. In contrast, our AdmixtureBayes, TreeMix, and OrientAGraph results can be easily and freely reproduced. Lastly, the OrientAGraph paper performs an excellent comparison of TreeMix, OrientAGraph, and miqograph. The authors find that indeed miqograph is unable to infer the correct admixture graph in all of the cases where there is an admixture event not producing a leaf node (M4 and M6 in Table 1 from that paper). For this reason, we think that adding a comparison with OrientAGraph and directing the reader towards the miqograph comparison from that paper is the best approach. We have added a sentence to the “Comparisons with TreeMix and OrientAGraph” section that highlights the comparison done in Molloy et al.

#3. Admixture Bayes Method

+3a. Nielsen et al (2021) write on page 3, "We here improve on these approaches by developing a novel MCMC sampling method, AdmixtureBayes, that can sample from the posterior distribution of admixture graphs. This enables an efficient search of the entire state space a well as the ability to report a level of confidence in the sampled graphs." I recommend replacing "efficient" with "effective" or some other adjective because this study at present doesn't emphasize efficiency. In particular, metrics like runtime are not currently reported for all methods / data sets studied. In addition, the authors do not report the number of admixture graph topologies explored by the different methods. My guess is that the MCMC method is less efficient because the runtime on the biological data set was 50 hours (my guess is that TreeMix

would take just a few minutes on this data set discounting the time to estimate summary statistics from allele frequencies).

We have replaced efficient with effective. You are correct that we do not prioritize runtime as a consideration.

+3b. I found the description of AdmixtureBayes to be well-written and well-motivated. My only questions was how the MCMC begins. The proposals must be performed on some starting admixture graph (both topology and numerical parameters), so I was curious whether this graph was selected at random or constructed using some heuristic, or something in between.

Thank you for bringing this omission to our attention. We have added a section titled "Initialization of AdmixtureBayes" to the appendix where we explain our construction of a random initial graph. We also justify this choice by appealing to assessing convergence through Gelman-Rubin statistics.

+3c. Nielsen et al (2021) define the concepts of minimal topology and consensus graphs, along with tools for computing and visualizing them. It would be great could if the authors discuss how these ideas compare to the techniques used by existing methods (like PhyloNet), which I believe also produce summarizes of network space.

Thank you for this comment. The concept of a topology set is indeed similar to PhyloNet's approach of considering the set of clusters induced by each edge of an evolutionary network. We have noted this similarity in the "Graph Summaries" section. As far as we are aware, the concept of a minimal topology induced by equivalence classes of topology sets and labeled with node persistence probabilities is novel, as is using the posterior distribution of certain nodes to generate a consensus graph. This is because these concepts are based on obtaining samples of admixture graphs from a specified probability distribution.

#5. Results

+5a. I found Figure S1/S2/S3/S14 to be somewhat difficult to interpret because only a single value is reported for multiple data sets. I recommend Nielsen et al (2021) re-plot these figures as boxplots (with data points plotted over the boxes) or as violin plots. It could also be helpful to plot topological error for TreeMix (e.g. set distance between true graph and estimated graph) against error for AdmixtureBayes (e.g., set distance between true graph and MAP graph). This way would cleary show when AdmixtureGraph is outperforming TreeMix for each data set. Alternatively, the difference between methods on a given data set could be plotted as box plot.

Thank you for this comment. We have done a major rewrite of the simulation study results. In our new simulation study, we evaluate the covariance distance, topology equality, and set distance for TreeMix, OrientAGraph, the Mode estimate obtained by AdmixtureBayes, and the mean graph sampled from the AdmixtureBayes posterior. We do this for 20 simulated datasets

for a set of 4 admixture graphs. We have plotted the results as boxplots when applicable. The topology equality for TreeMix, OrientAGraph, and the AdmixtureBayes Mode estimate are all either 0 or 1, so we find it is not informative to plot a boxplot in these cases. We instead plot a single value at the mean across the 20 simulations.

+5b. Currently, the authors provide a text description of differences between prior analyses on the "peopling of the Americas" and the results of running AdmixtureBayes. It would be very helpful to show this information as a figure. For example, it would be great if Nielsen et al (2021) provided the result of running TreeMix as part of Figure S4, with the same node labelings.

Thank you for this suggestion. We have added a new figure where we run TreeMix and OrientAGraph on our dataset for varying numbers of admixture events.

+5c. I recommend the authors compute likelihood scores for the graphs shown in Figures S4, S5, and S6 and include this information in the figure (it seems like numerical parameters could simply be reoptimized for the given topologies and then the likelihood scores reported). This information would help readers compare the results to prior studies where likelihood scores have been reported. It could also emphasize points being made by Nielsen et al (2021) about the values of Bayesian methods compared to ML methods in this context.

Thank you for this comment. We do indeed compute and report the log likelihood for each graph we sample. In particular, our output for Step 1 is a csv of sampled graphs. Each line represents one graph in the output file. Within each row there are columns related to the representation of the tree, but there are also many statistics that might be of interest to the user. These include the log likelihood, the log prior, the log posterior, the number of admixture events, and the total branch length of the tree. We initially wanted to print these values in order for the user to be able to assess convergence, but you are correct that another use could be reoptimizing the continuous parameters of a given admixture graph. We have made this clear in the GitHub documentation and explained the relevant columns of the output file.

+5d. I recommend Nielsen et al (2021) provide the specific commands used for running TreeMix and AdmixtureBayes be provided (or provide the analysis scripts).

This is a good recommendation. We have uploaded all relevant simulation scripts and AdmixtureBayes, TreeMix, and OrientAGraph commands to the AdmixtureBayes GitHub. We have also listed the name of the relevant GitHub location in the manuscript where appropriate.
